# Mechanical Aortic Valve Replacement Following Self-Inflicted Cardiac Needle Penetration in a Patient with Recurrent Self-Harm: A Case Report with Fatal Long-Term Outcome

**DOI:** 10.3390/jcm15124544

**Published:** 2026-06-11

**Authors:** Martin Breitwieser, Philipp Krombholz-Reindl, Georg Hattwich, Thomas Freude, Marian Mitterer

**Affiliations:** 1 Department for Orthopedic Surgery and Traumatology, Paracelsus Medical University Hospital Salzburg, 5020 Salzburg, Austria; 2 Department of Vascular and Endovascular Surgery, Paracelsus Medical University Hospital Salzburg, 5020 Salzburg, Austria; 3 Department of Cardiac Surgery, Paracelsus Medical University Hospital Salzburg, 5020 Salzburg, Austria; 4 Department of Psychiatry and Psychotherapy, Paracelsus Medical University Hospital Salzburg, 5020 Salzburg, Austria

**Keywords:** mechanical aortic valve, self-inflicted injury, cardiac foreign body, anticoagulation adherence, psychiatric disorder, prosthetic valve thrombosis

## Abstract

**Background and Clinical Significance**: We report an exceptionally rare case of mechanical aortic valve replacement necessitated by self-inflicted needle penetration with aortic valve and left ventricular involvement in a patient with recurrent self-harm behavior. **Case Presentation**: A 24-year-old female with post-traumatic stress disorder and emotionally unstable personality disorder, borderline type, presented with dyspnea two weeks after self-inserting multiple needles into her thorax. Computed tomography revealed a needle lodged in the aortic root and an intramyocardial needle with hemorrhagic pericardial effusion. Emergency sternotomy revealed inflammatory destruction of the right coronary aortic cusp with complete perforation. Following failed reconstruction attempts, mechanical aortic valve replacement was performed. The patient survived the immediate postoperative period but demonstrated recurrent non-adherence to oral anticoagulation, including multiple episodes of over- and under-anticoagulation. More than six years after surgery, she presented with cardiogenic shock due to prosthetic valve thrombosis after discontinuing warfarin for two weeks. Despite venoarterial ECMO and fibrinolytic therapy, she died from refractory left ventricular failure. **Conclusions**: This case highlights critical challenges in managing patients with severe psychiatric disorders requiring mechanical valve prostheses and suggests that bioprosthetic valves may warrant careful consideration in patients with major concerns regarding long-term anticoagulation adherence.

## 1. Introduction and Clinical Significance

Self-inflicted cardiac injuries with needles represent a rare but potentially life-threatening manifestation of severe psychiatric disorders, predominantly occurring in patients with depression, schizophrenia, borderline personality disorder, post-traumatic stress disorder (PTSD), and substance abuse [1,2,3]. Since the comprehensive review by Schechter and Gilbert in 1969 [4], which documented 157 cases of cardiac injuries from pins and needles, approximately 40 additional cases have been reported in the English literature with a marked female predominance (65–70%) and median age of 29–36 years [5]. The majority of reported patients (85%) required surgical intervention, with overall mortality of approximately 5% when treated surgically [3,6].

Reported complications include cardiac tamponade (15–20%), mural thrombus formation (10%), valve dysfunction (rare), peripheral embolism (5%), and chronic constrictive pericarditis [7,8,9]. While most published cases involve simple needle extraction from the myocardium or pericardium [10,11], aortic valve involvement requiring replacement has not been previously described.

Recurrent self-harm with cardiac needle insertion is exceptionally rare. Sadeghi et al. reported a 29-year-old male with bipolar disorder who required two sternotomies within two years [12]. A 17-year-old female inserted needles twice within two months [5]. Our patient represents the most severe reported case with three escalating episodes over seven months, culminating in aortic valve destruction and mechanical prosthetic heart valve reconstruction.

These mechanical prosthetic heart valves require lifelong anticoagulation with vitamin K antagonists to prevent thromboembolism, with an annual risk of valve thrombosis of 0.1–5.7% even with adequate anticoagulation [13,14]. Poor anticoagulation control, defined as a time in therapeutic range (TTR) < 60%, significantly increases the risk of both thrombotic and hemorrhagic complications [15]. In patients with psychiatric disorders and potential adherence issues, the choice between mechanical and bioprosthetic valves poses a significant clinical dilemma and specific recommendations are lacking in the literature.

We present a case of mechanical aortic valve replacement following self-inflicted cardiac needle injury in a young woman with recurrent self-harm behavior, with more than six years of follow-up demonstrating fatal consequences of anticoagulation non-adherence. This case provides important insights into valve selection criteria in psychiatrically complex patients and interdisciplinary management challenges between cardio-thoracic surgery, internal and intensive care medicine, psychiatry and trauma surgery.

## 2. Case Presentation

### 2.1. Patient History and Initial Presentation

A 24-year-old female with type 2 diabetes mellitus and a psychiatric history later characterized as post-traumatic stress disorder and emotionally unstable personality disorder presented to the trauma surgery emergency department in March 2018 with dyspnea and retrosternal chest pain two weeks after self-inserting 6–8 diabetic lancet needles into the left anterior chest wall. Before 2017, no psychiatric or psychotherapeutic treatment had been documented; later psychiatric evaluation revealed childhood physical abuse, two prior suicide attempts, chronic suicidal ideation, and no structural cerebral abnormalities on cranial CT. The March 2018 presentation represented the third episode of escalating self-injurious needle insertion within seven months, after previous insertions into the left forearm and cervical region, with only partial removal of the cervical needles possible because of vascular and neural risk.

### 2.2. Clinical Examination and Imaging

On presentation, the patient was awake, oriented, mildly dyspneic, hemodynamically stable, and afebrile, without visible entry wounds or clinical signs of infection. Chest radiography demonstrated multiple radiopaque needle-shaped foreign bodies throughout the thorax (Figure 1).

Contrast-enhanced CT of the neck and thorax showed multiple retained and newly inserted cervical, subclavian, pectoral, thoracic, and intra-abdominal needles, with one needle embedded in the aortic root and another penetrating the posterolateral apical left ventricular myocardium; a circumferential hyperdense pericardial effusion consistent with hemopericardium was also present (Figure 2).

Transthoracic echocardiography demonstrated hemodynamically significant pericardial effusion with right atrial compression, preserved left ventricular function, markedly elevated aortic valve gradients, and severe aortic insufficiency, with suspected perforation of the right coronary cusp by the needle lodged in the aortic root.

### 2.3. Surgical Intervention

Preoperatively, informed consent was obtained for emergency surgical removal of the intracardiac foreign bodies and treatment of associated cardiac injury. Valve replacement was not planned primarily, but was discussed as a possible intraoperative emergency measure if repair proved insufficient.

Median sternotomy was performed. Needles located in the presternal and lateral thoracic wall tissues could not be localized despite C-arm fluoroscopy because of their small diameter and were therefore left in situ.

Upon pericardiotomy, hemorrhagic fluid was evacuated, the heart was carefully inspected, and the intramyocardial needle was identified penetrating the diaphragmatic wall of the left ventricle near the apex (Figure 3A). After luxation of the heart, the needle was extracted (Figure 3B) and the myocardial puncture site was oversewn with Prolene 4–0 suture.

The patient was placed on cardiopulmonary bypass via aortic and right atrial cannulation. An antegrade cardioplegia line was inserted and the aorta was cross-clamped. Transverse aortotomy revealed the second needle traversing the right coronary cusp of the aortic valve, with marked inflammatory changes and complete cusp perforation resulting in severe aortic regurgitation (Figure 3C).

#### 2.3.1. Initial Reconstruction Attempt

Because of the patient’s young age and concerns regarding lifelong anticoagulation adherence in the setting of recurrent self-harm, valve-sparing reconstruction with a glutaraldehyde-fixed pericardial patch was attempted after needle removal and debridement of inflamed cusp tissue, but relevant aortic insufficiency persisted despite revision of the repair.

#### 2.3.2. Definitive Mechanical Valve Replacement

After interdisciplinary intraoperative discussion and failed reconstruction, the remaining native cusps were excised and a 21 mm On-X mechanical bileaflet aortic valve prosthesis was implanted, followed by successful separation from cardiopulmonary bypass and transfer to the ICU in hemodynamically stable condition.

### 2.4. Postoperative Course

Postoperatively, empiric antibiotic therapy with flucloxacillin, ampicillin, and gentamicin was administered for 10 days as prophylactic coverage in the setting of traumatic intracardiac foreign-body injury and inflammatory valve destruction; valve tissue cultures, PCR analysis, and intraoperative blood cultures remained negative. The patient was extubated on postoperative day 1, transferred to the cardio-thoracic surgery ward on postoperative day 3, and serial echocardiography confirmed normal prosthetic valve function without paravalvular leak or aortic insufficiency.

Repeated liaison psychiatric assessments documented no florid psychosis, no severe formal thought disorder, and no acute suicidality, although inpatient psychiatric treatment was recommended because of the recurrent self-harm trajectory and was declined by the patient.

Warfarin therapy was initiated with low-molecular-weight heparin bridging, and extensive patient and family education was provided regarding the necessity of lifelong anticoagulation and the potentially fatal consequences of warfarin discontinuation. She was discharged on postoperative day 15 in good condition, with documented insight into the need for anticoagulation but persistent concern regarding long-term treatment reliability.

### 2.5. Long-Term Follow-Up and Recurrent Complications

Following discharge, the patient initially recovered well from a cardio-thoracic surgical perspective, with no immediate prosthesis-related complications and satisfactory valve function. Over the subsequent six years and four months, however, she experienced recurrent self-harm, intentional warfarin overdose requiring psychiatric hospitalization, repeated severe over-anticoagulation, hemorrhagic complications, inflammatory complications related to retained cervical needles, and infectious-urological complications in the setting of unstable anticoagulation. Although intermittent psychosocial stabilization and ongoing psychotherapy were documented, long-term treatment reliability remained limited.

### 2.6. Fatal Presentation

In July 2024, six years and four months after mechanical aortic valve replacement, the patient presented to the emergency department with progressive dyspnea and interscapular chest pain for one week. She reported having discontinued warfarin for approximately two weeks because she was unable to locate her medication. She denied suicidal intent in relation to this interruption.

Initial vital signs revealed tachycardia (140 bpm), normotension (systolic > 110 mmHg), and increasing respiratory distress. Physical examination demonstrated cold, clammy extremities and signs of cardiogenic shock. Laboratory studies revealed markedly elevated troponin (25,000 ng/L), elevated lactate (progressing to 10 mmol/L), INR 1.0 and hemoglobin 12.9 g/dL.

While undergoing emergency CT aortography, the patient developed severe respiratory failure and shortly thereafter suffered cardiac arrest with pulseless electrical activity.

Advanced cardiac life support was initiated immediately. After 45 min of high-quality cardiopulmonary resuscitation under mechanical chest compression, emergency venoarterial extracorporeal membrane oxygenation (VA-ECMO) was established via femoral cannulation (right femoral vein and left femoral artery) with distal perfusion cannula placement.

### 2.7. Intensive Care Management

The patient was transferred to the cardio-thoracic intensive care unit on VA-ECMO (flow 4.7 L/min) with extremely high vasopressor requirements (norepinephrine 1 μg/kg/min, vasopressin 4 units/hour). Initial lactate was 8.7 mmol/L with metabolic acidosis. She remained intubated, sedated, and invasively monitored.

Given the patient’s critical condition and severe left ventricular dysfunction from both the ischemic insult during arrest and the marked left ventricular dilatation from aortic insufficiency and retrograde ECMO flow, an interdisciplinary team (cardio-thoracic surgery, cardiology, and intensive care) concluded she was not a surgical candidate for reoperation. Additionally, she was considered unsuitable for ventricular assist device implantation or heart transplantation due to active severe psychiatric illness with recurrent suicidal behavior and demonstrated inability to maintain medication adherence.

Fibrinolytic therapy was therefore pursued in accordance with current guideline recommendations for prosthetic valve thrombosis [16]. An initial regimen of recombinant tissue plasminogen activator (alteplase; 10 mg bolus followed by 90 mg over 90 min) produced no improvement on post-lysis transesophageal echocardiography, which continued to show persistent thrombus and unchanged ventricular function. As a final salvage measure, continuous urokinase infusion (50,000–100,000 units/hour for 24 h) was subsequently administered, again without improvement in leaflet mobility or left ventricular function. The patient remained dependent on maximal vasopressor support and developed progressive renal and multi-organ failure.

### 2.8. Family Conference and Therapy Withdrawal

Multiple family conferences were held with the patient’s mother, siblings, and partner over the next 3 days after initial presentation to the ED and CPR. After extensive discussion and with family agreement, therapy was redirected to comfort-focused care. Vasopressors and ECMO support were withdrawn. The patient died peacefully in the presence of her family, six years and four months after mechanical aortic valve implantation.

## 3. Discussion

This case illustrates the central dilemma of prosthesis selection after self-inflicted cardiac injury in a young patient with recurrent self-harm and uncertain long-term treatment reliability. The immediate surgical priority was to remove the intracardiac foreign bodies, treat hemopericardium, and restore aortic valve competence. Valve-sparing reconstruction was therefore attempted first, because the patient was only 24 years old and lifelong anticoagulation was already a major concern in the context of psychiatric instability. However, persistent grade II aortic insufficiency after two repair attempts made definitive valve replacement necessary.

The key decision was therefore not whether the damaged valve required definitive treatment, but which prosthesis type best matched the patient’s long-term risk profile. A mechanical valve offered durability and was consistent with usual age-based considerations in young patients. At the same time, it imposed a lifelong requirement for reliable vitamin K antagonist therapy. A biological prosthesis would likely have reduced the risk of fatal prosthetic valve thrombosis related to warfarin discontinuation, but would have exposed the patient to the expected risk of structural valve degeneration and future reintervention at a young age. The available documentation does not suggest that the full extent of future anticoagulation instability could have been anticipated at the time of surgery.

In retrospect, the fatal outcome after warfarin discontinuation more than six years later suggests that a bioprosthetic valve might have been a reasonable alternative despite the patient’s young age. However, this case should not be interpreted as a general recommendation against mechanical valves in patients with psychiatric illness. Rather, it suggests that in selected younger patients with recurrent self-harm, marked psychiatric instability, or major concern regarding long-term anticoagulation adherence, prosthesis selection should explicitly include the question of whether the long-term treatment demands of a mechanical valve are realistically compatible with the patient’s expected treatment reliability.

## 4. Conclusions

We present an exceptionally rare case of mechanical aortic valve replacement necessitated by self-inflicted needle injury with aortic valve and left ventricular penetration in a young woman with recurrent self-harm. Despite successful surgery and initial recovery, the patient demonstrated catastrophic anticoagulation non-adherence over more than six years, culminating in fatal prosthetic valve thrombosis.

This case highlights three important lessons. First, self-inflicted cardiac injury requires close interdisciplinary management involving cardio-thoracic surgery, trauma surgery, intensive care, and psychiatry. Second, short-term postoperative stability does not necessarily predict long-term treatment reliability in patients with recurrent self-harm and chronic psychiatric vulnerability. Third, in selected younger patients with major concerns regarding long-term anticoagulation adherence, bioprosthetic valve replacement may warrant careful consideration despite the usual durability advantages of mechanical prostheses.

## Figures and Tables

**Figure 1:**
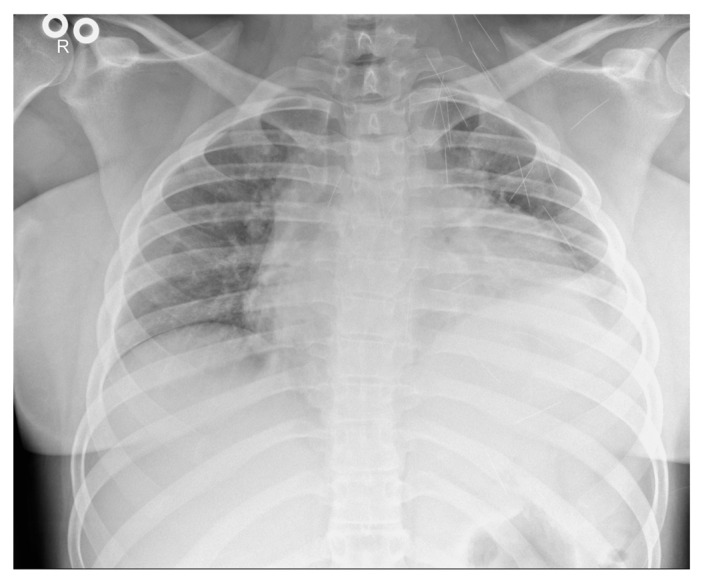
**Preoperative Chest Radiograph.** Anteroposterior view demonstrating multiple radiopaque needle-shaped foreign bodies distributed throughout the left hemithorax and mediastinum.

**Figure 2:**
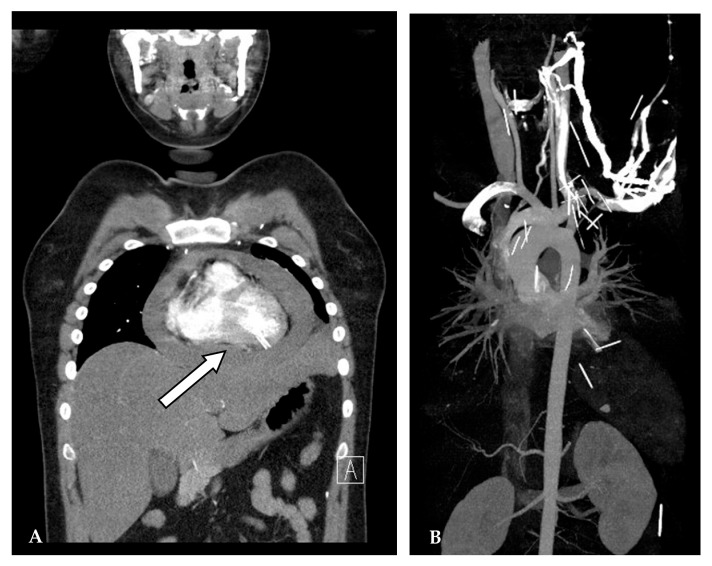
**Preoperative CT scan.** (**A**) Coronal CT section demonstrating a needle penetrating the left ventricular myocardium (arrow) with a large hemorrhagic pericardial effusion. (**B**) Three-dimensional volume-rendered CT reconstruction showing spatial distribution of self-inserted needles in relation to the heart and great vessels.

**Figure 3:**
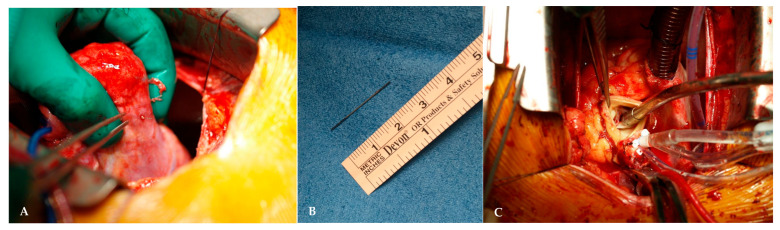
**Intraoperative Findings.** (**A**) Metallic needle penetrating the left ventricular myocardium near the apex after pericardiotomy. (**B**) Extracted intramyocardial needle (diabetic lancet, approximately 3 cm in length). (**C**) Needle traversing the right coronary aortic valve cusp after aortotomy, showing cusp perforation.

## Data Availability

The data are not publicly available due to patient privacy and ethical restrictions. All relevant clinical information has been included in the published case report.

## Abbreviations

The following abbreviations are used in this manuscript:

bpm	Beats per minute
CPR	Cardiopulmonary resuscitation
CT	Computed tomography
ECMO	Extracorporeal membrane oxygenation
EACTS	European Association for Cardio-Thoracic Surgery
EF	Ejection fraction
ESC	European Society of Cardiology
ICU	Intensive care unit
INR	International normalized ratio
LMWH	Low-molecular-weight heparin
MVT	Mechanical valve thrombosis
PCR	Polymerase chain reaction
PTSD	Post-traumatic stress disorder
PTZ	Prothrombin time percentage
TEE	Transesophageal echocardiography
TTR	Time in therapeutic range
VA-ECMO	Venoarterial extracorporeal membrane oxygenation
VKA	Vitamin K antagonist
Vmax	Peak maximum velocity
